# Predicting Water Cycle Characteristics from Percolation Theory and Observational Data

**DOI:** 10.3390/ijerph17030734

**Published:** 2020-01-23

**Authors:** Allen Hunt, Boris Faybishenko, Behzad Ghanbarian, Markus Egli, Fang Yu

**Affiliations:** 1 Department of Physics and Department of Earth & Environmental Sciences, Wright State University, 3640 Colonel Glenn Highway, Dayton, OH 45435, USA; 2 Energy Geosciences Division, E. O. Lawrence Berkeley National Laboratory, University of California, 1 Cyclotron Rd., Berkeley, CA 94720, USA; bafaybishenko@lbl.gov; 3 Porous Media Research Lab, Department of Geology, Kansas State University, Manhattan, KS 66506, USA; ghanbarian@ksu.edu; 4 Department of Geography, University of Zürich, 8057 Zürich, Switzerland; markegli@bluewin.ch; 5 Department of Forestry, Beihua University, 3999 Binjiangdong Road, Jilin 132013, China; yu.39@wright.edu

**Keywords:** subsurface hydrology, percolation theory, solute transport, chemical weathering, water balance

## Abstract

The fate of water and water-soluble toxic wastes in the subsurface is of high importance for many scientific and practical applications. Although solute transport is proportional to water flow rates, theoretical and experimental studies show that heavy-tailed (power-law) solute transport distribution can cause chemical transport retardation, prolonging clean-up time-scales greatly. However, no consensus exists as to the physical basis of such transport laws. In percolation theory, the scaling behavior of such transport rarely relates to specific medium characteristics, but strongly to the dimensionality of the connectivity of the flow paths (for example, two- or three-dimensional, as in fractured-porous media or heterogeneous sediments), as well as to the saturation characteristics (i.e., wetting, drying, and entrapped air). In accordance with the proposed relevance of percolation models of solute transport to environmental clean-up, these predictions also prove relevant to transport-limited chemical weathering and soil formation, where the heavy-tailed distributions slow chemical weathering over time. The predictions of percolation theory have been tested in laboratory and field experiments on reactive solute transport, chemical weathering, and soil formation and found accurate. Recently, this theoretical framework has also been applied to the water partitioning at the Earth’s surface between evapotranspiration, *ET*, and run-off, *Q*, known as the water balance. A well-known phenomenological model by Budyko addressed the relationship between the ratio of the actual evapotranspiration (*ET*) and precipitation, *ET/P*, versus the aridity index, *ET_0_/P*, with *P* being the precipitation and *ET_0_* being the potential evapotranspiration. Existing work was able to predict the global fractions of *P* represented by *Q* and *ET* through an optimization of plant productivity, in which downward water fluxes affect soil depth, and upward fluxes plant growth. In the present work, based likewise on the concepts of percolation theory, we extend Budyko’s model, and address the partitioning of run-off Q into its surface and subsurface components, as well as the contribution of interception to *ET*. Using various published data sources on the magnitudes of interception and information regarding the partitioning of *Q*, we address the variability in *ET* resulting from these processes. The global success of this prediction demonstrated here provides additional support for the universal applicability of percolation theory for solute transport as well as guidance in predicting the component of subsurface run-off, important for predicting natural flow rates through contaminated aquifers.

## 1. Introduction and General Background

The fate of water falling on the surface of the Earth as precipitation is the central issue of hydrologic sciences [1,2,3,4,5,6]. The partitioning of water between its various components and pathways is crucial to processes such as soil erosion, soil production, and vegetation growth and productivity, as well as flooding and water resources. The hydrologic cycle interference by humans is high due to the competition of all manner of demands on water as well as the desire to utilize the Earth’s surface to its fullest potential. In keeping with requirements of efficiency, intervention, in the sense of irrigation and impoundment, peaks downstream, when flow is focused. The water cycle represents simultaneously a critical input to human society as well as to the sciences of geology, ecology, and global biogeochemical cycles [7].

Water paths at the Earth’s surface are intimately linked with evapotranspiration, infiltration, and run-off, as well as with the subsurface–atmospheric carbon cycle. Water that flows into the subsurface drives the weathering of topsoil, subsurface sediments, and underlying bedrock. Weathering rates are limited chiefly by the ability of water to transport weathering products from the weathering front through the subsurface into streams (e.g., [8,9,10,11]). There is a strong analogy between this transport process and the ability of water to transport contaminants away from their subsurface concentrations. Weathering of the dominant (mainly, silicate) minerals on the Earth’s surface through interaction with dissolved CO_2_ and organic acids represents by far the largest sink for atmospheric carbon [12]. In contrast, [13] hypothesized that the silicate weathering’s share to atmospheric CO_2_ sink has been overestimated, indicating the presence of other sources of atmospheric CO_2_. Due to rapid kinetics, carbonate weathering in the short term might be significant in controlling the climate (even though the net drawdown by this pathway is zero). Vegetation growth and production of plant material represents a much more rapidly varying sink for atmospheric carbon, although with far less total sequestration [12]. Weathering of rocks to their products typically enables physical erosion as well [14,15,16,17,18] thus placing constraints on the rock mineralogical cycle, deep water cycle, and deep carbon cycle. Water importance in all these processes should not be underestimated. Since the rate of each of these processes depends on the magnitude of the associated water fluxes [19,20,21,22,23], understanding the partitioning of water into these fundamental fluxes will help future modeling efforts to generate reliable predictions of both these fluxes and their associated processes. In an operational perspective, the soil–water balance can be represented simply in terms of a mass conservation law. However, it is desirable to determine what fraction of the precipitation follows each specific pathway through the environment. A more challenging problem is to predict how these precipitation fractions depend on climate along various gradients. Such a prediction may be adaptable to employment in climate change models as well as to understanding current results.

The basis of predictions of solute transport through the soil is the evaluation of the water balance, both affecting abiotically the process of chemical weathering and associated soil formation [24,25], and biotically, the process of plant growth. Incorporating the effects of these processes, which depend on the run-off, *Q*, and the evapotranspirative water flux, *ET*, respectively, allows one to optimize the net primary productivity (*NPP*) of an ecosystem with respect to *ET*. For example, in [26], the globally-averaged value of *ET* was predicted as a fraction of *P*. In addition, in [27] further strong support was provided for the use of universal principles of percolation theory for predictions of solute transport.

## 2. Objectives

To understand the magnitudes of *Q* and *ET*, including both their variability at a given aridity index and their dependence on aridity index, the goal of current publication is to address the surface and subsurface components of *Q* and the relative contributions of interception and transpiration for *ET*.

A previous publication, [26] by one of us addressed the partitioning of *ET* into transpiration and evaporation off bare ground for *ET*_0_/*P* ≫ 1, while, in the current paper, we address partitioning of *ET* into evaporation and transpiration for *ET*_0_/*P* ≪ 1. Division into components of evaporation and surface run-off, which do not intersect the carbon cycle meaningfully, and transpiration and subsurface run-off, which do, would make our treatment more compatible with research into the carbon cycle and its interactions with the water cycle under various scenarios of climate change.

In the following, we first summarize the basic arguments that led to a solution for *ET*/*P* and generated a result in accordance with the global mean value. Then, a brief overview of how that solution was extended to generate *ET*/*P* for any range of *ET*_0_/*P* values together with a short discussion of how the solution can be extended to give a predicted range of *ET*/*P* values for any particular value of the *ET*_0_/*P* parameter. Next, we present the extension to address the partitioning of *Q* as well as *ET*.

## 3. Budyko Theory

Budyko [28,29] noted certain formal similarities between the water budget and the energy budget. Based on these ideas, Budyko then proposed a potential analytical solution that should give the fraction of water that is returned to the atmosphere as *ET* as a function of a ratio of incident solar energy to precipitation. However, to establish the analogies that led to the solution, Budyko had to simplify the problem, lumping both physical evaporation and plant transpiration into a single variable, evapotranspiration (*ET*), and surface and subsurface run-off into the quantity run-off (*Q*).

Budyko’s [28,29] formulation is the starting point for the present work. In contrast to a previous effort [26], which addressed primarily the variation of *ET* as a function of climate and then the variability of *ET* at a given climate index, we investigate here an expansion of the technique to allow for separate treatments of surface and subsurface run-off as well as the partitioning of evapotranspiration into evaporation and transpiration. Consequently, we also have to extend the fundamental equation for the water balance that was treated by [28,29]. Many possible such extensions could be formulated, depending on how finely the distinctions between the various water paths are examined. 

In Budyko’s simple form (all quantities given as rates), the water balance is represented by,

(1) $$P=Q+ET+\frac{dS}{dt}$$

where *P* is the precipitation and *dS*/*dt* is the rate of change of water storage in the subsurface. Typically, such quantities are measured or inferred over times of years or longer. It is common to consider (i.e., average over) a period of time sufficiently long that ⟨*dS*/*dt*〉 can be assumed to be zero. Here, the brackets ⟨ 〉 signify temporal averaging. Setting ⟨*dS*/*dt*〉 = 0 yields,

⟨*P*〉 = ⟨*Q*〉 + ⟨*ET*〉 (2)

The assumption that ⟨*dS*/*dt*〉 = 0 will likely be inaccurate over relatively short time periods, which may introduce some uncertainties into predictions and a comparison of theory and observations. For example, when significant extraction of fossil (e.g., Pleistocene) groundwater reserves is utilized for agriculture in areas where climate in the Holocene has become significantly more arid, ⟨*dS*/*dt*〉 will be negative and non-negligible. Otherwise, it was assumed [30] that neglecting ⟨*dS*/*dt*〉 requires only considerable time to be reasonably accurate. According to those authors, although twenty years is not sufficient to smooth out fluctuations in storage, a 50-year record is normally adequate. Climate change, if rapid, could still complicate establishment of such a criterion.

What values of the time period(s) should one adopt for calculations of ⟨*ET*〉 and ⟨*Q*〉? This depends on the physical location and climatic conditions. We are interested first in the global average.

“In the continental United States, approximately 2/3 of all rainfall delivered is lost to evapotranspiration” [31,32]. Yin et al. [33] however, “show that North American Regional Reanalysis data overestimate surface evaporation”. Recent results for global ⟨*ET*〉 [34,35] give, respectively, 0.639 and 0.634. “Over 61% of rainfall is lost to evapotranspiration, so mapping its variance is an important part of understanding the global water cycle.” [36]. Other estimates of terrestrial *ET*/*P* are about 59% [37], 61% [38,39,40], and 67% [41]. A simple mean of these eight global results (assuming “over 61%” = 62%) yields *ET*/*P* = 0.623 ± 0.025. An earlier meta-study [42], which surveyed six published results [28,43,44,45,46,47] generated a mean *ET*/*P* = 0.645 ± 0.044. The overall mean of all 14 studies is *ET*/*P* = 0.632; either way, the fraction of *P* returned to the atmosphere by evaporation is significantly larger than the fraction going to run-off, although the trend in the data makes it look as though the fraction is less than 2/3 and declining. Jung and Reichstein [48] in fact, suggested that ⟨*ET*〉 as a function of ⟨*P*〉 has been varying since the 1970s and has more recently been declining due to depletion of soil moisture. Although the two quoted meta-results appear to support such a decline, in fact, they do not differ statistically, considering that the small difference in means (0.022) lies within the standard deviation of either. Further discussion requires introduction of the parallel concept of an energy balance.

The radiation budget at the terrestrial Earth’s surface is written in a similar fashion to the water. In steady-state, what incident radiation (*R*) cannot be accommodated through the evaporation of water is transmitted away from the surface as heat, *H*.

(3) R=ET·L+H

where *R* is the radiant energy incident on the surface and *L* is the latent heat of water. We should point out that neither long-term changes in the surface temperature nor melting of constituents (such as ice and glaciers) are accounted for in Equation (3). Ignoring such change is equivalent to setting yearly and multi-decadal averages of the radiative variables equal. Thus, while a distinction between yearly values of the radiative variables and their long-term averages could be made, it is of little significance as long as climate change is not addressed explicitly. Division by *L* leads to,

(4) $$\frac{R}{L}\equiv ET_{0}=ET+\frac{H}{L}$$

In Equation (4), the quantity *ET*_0_ is known as the potential evapotranspiration and corresponds to how much water could be evaporated if all the radiant energy falling on the Earth’s surface caused water evaporation. If *ET*_0_ = ⟨*P*〉, it is possible for all the precipitation falling to the Earth to be evaporated and simultaneously for all radiant energy to be consumed in the process of evaporating water. Thus, both *H* in Equation (4) and ⟨*Q*〉 in Equation (3) could be zero. Such an idealization is, of course, never realized, as it would require *ET*_0_ = ⟨*P*〉 in essentially every time period. With an increase in ⟨*P*〉 (or decrease in *ET*_0_), ⟨*Q*〉 must be non-zero. This condition is known as energy-limited. With a decrease in ⟨*P*〉 (or increase in *ET*_0_), *H*/*L* must be non-zero. The corresponding condition is referred to as water-limited.

In arid climates, for which *ET*_0_/⟨*P*〉 ≫ 1, there may be far less water available for evaporation than could actually be evaporated. *ET*_0_/⟨*P*〉 is accordingly known as the aridity index. Thus, for large aridity index, *H*/*L* in Equation (4) is likely to be large and ⟨*Q*〉 near zero, whereas, in the limit that the aridity index is much smaller than 1, *H*/*L* is likely to be near zero and *Q* large.

The similarity between Equations (4) and (2) spurred Budyko to a universal formulation for the quantity ⟨*ET*/*P*〉 as a function of *ET*_0_/⟨*P*〉. While the functional form cannot be rigorously derived by any procedure, many models have been constructed that lead to results similar, or equal, to the Budyko phenomenological result. This interest owes to the relatively good job that the phenomenology does in predicting actual values of ⟨*ET*〉/⟨*P*〉 as a fraction of *ET*_0_/⟨*P*〉, meaning that the relationship that Budyko proposed can be used as a first estimate for ecosystems and drainage basins with unmeasured values of ⟨*ET*〉/⟨*P*〉. Moreover, Budyko [28] also found reasonable limits for ⟨*ET*〉/⟨*P*〉 in the extreme cases of water-limited ecosystems (*ET*_0_/⟨*P*〉 ≫ 1) and energy-limited ecosystems (*ET*_0_/⟨*P*〉 ≪ 1). In the former case, ⟨*ET*〉/⟨*P*〉 must approach 1, whereas, in the latter, ⟨*ET*〉/⟨*P*〉 should not exceed *ET*_0_/⟨*P*〉. The dependence of ⟨*ET*〉/⟨*P*〉 on the aridity index, *ET*_0_/⟨*P*〉, has elicited significant attention in the hydrologic sciences [49,50], and a wide variety of means to address this problem have been proposed [2,3,49,51,52,53,54,55].

## 4. Data Sources

Previously, Hunt [26] compared prediction of ⟨*ET*〉/⟨*P*〉 with long-term (50 years or greater) records of evapotranspiration in individual basins. These data were compiled by [30] and divided into three categories: “out of phase”, when the maximum in precipitation does not occur at the same time as the maximum in irradiance (temperate climate zones); “in phase”, appropriate for tropical regions; and “non-seasonal”, for high maritime influence. Values chosen to represent the variability of plant root fractal dimensionality were taken from [56], who measured the root fractal dimensionality of 55 species of forbs and grasses that grow on the Great Plains of the USA. The same basic data sources are cited again here. However, we now address interception and the distinction between subsurface and surface run-off, requiring reference to a suite of results [57,58,59,60,61,62] for the magnitude of the interception term, as well as a comprehensive review [63] on the relative contributions of surface and sub-surface run-off to *Q*.

## 5. Concepts from Percolation Theory

The basic components of the theoretical framework underlying the current research relate to plant growth and soil formation. First, the spatiotemporal scaling relationships for these two processes and then the actual length and time scales are given to complete the relationships [22].

**Figure 1:**
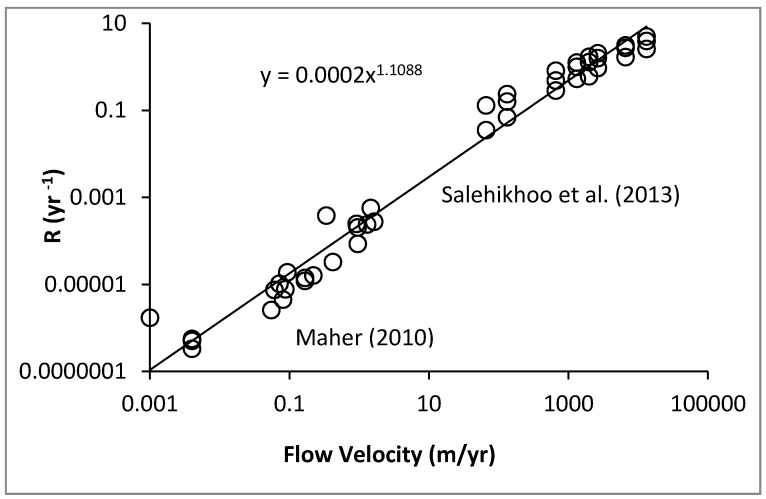
Rates of weathering in the lab [65] and in the field [21] as a function of throughflow. Field measurements were of silicates. Lab experiments were performed on magnesite, MgCO_3_, whose rapid reaction kinetics allows the approximately linear dependence on flow rate to extend to higher flow rates than in silicates, whose kinetic limitations are orders of magnitude smaller.

Theory, experimental results, and field observation all relate soil development and plant growth to the fundamental water fluxes. In the last decade, for example, it has become clear that silicate weathering processes in the field are largely subsurface flow-limited [21]. Moreover, by its compatibility with the field data, laboratory evidence (e.g., [64]) supports this assertion. Combining results from these two studies demonstrates a linear dependence of weathering rates on water flow rates over seven orders of magnitude of water flow rate, from field to the laboratory (Figure 1). However, there is an at least six orders of magnitude reduction in weathering rates over time scales from months to 5 Myr [65].

The correlation between transpiration and growth rates is less thoroughly exposited, but is demonstrated in [22,66] and is also known from a compilation of the world’s tallest trees [67]. The close correlation between mean values of *NPP* and *ET* is better documented (e.g., [19,68]).

In the case of woody plant growth, it is found that root radial extent *RRE* increases more slowly than root length on account of the tortuosity of the roots [56,69]. This is expressed using the tortuosity of optimal paths [70] through disordered two-dimensional networks, which relates actual distance along a tortuous path to Euclidean separation by the exponent 1.21 [71]. Thus, root length, *RL*, is predicted to relate to root radial extent through the power law, i.e., *RL* ∝ *RRE* ^1.21^. When root tip extension rates remain constant, the rate of lengthening of RRE diminishes in time. The preference for the two-dimensional context for selecting the optimal paths exponent is the relative shallow depth of tree roots in forests, often assumed and/or documented to be on the order of 1 m [72,73], or even less [30,74], on account of the shallow depth of most nutrient sources (Lynch, 1995). For length scales of 1–40 m, *RRE* and tree height are nearly identical [75,76,77]. Thus, slowing of tree growth in time is due to a reduction (in time) in the rate of access of nutrients from root growth along optimal flow paths.

In the case of soil production, soil depth is limited by chemical weathering, itself controlled by solute transport and mainly by the mechanism of advection. The solute transport, which is non-Gaussian (or non-Fickian, e.g., [78]), is proposed to follow the spatiotemporal scaling of percolation theory, governed by flow nominally perpendicular to the (thin, i.e., about 0.5–1.5 m thick) soil layer, thus in three dimensions. In particular, the time, *t*, for solute to be transported a Euclidean distance *x* is not *t* ∝ *x* (typical of advective solute transport as analyzed with the Advection–Dispersion Equation, ADE), but rather, *t* ∝ *x* ^1.87^. Thus, the travel distance after a time *t*, x (*t*), is proportional to *t* ^1/1.87^. The important consequence is that the solute velocity, *v*_s_, diminishes in time according to another power law, i.e., vs. ∝ *t* ^−0.87/1.87^. Both the soil production rate and the chemical weathering rate are then proportional to the solute velocity [79]. The high degree of universality of percolation exponents makes the results applicable for most systems at most length and time scales [27].

Finding the exponent of a power-law relationship is important, but it is important also to find a rate that relates the fundamental length scale of the system, *x*_0_, to a time scale, *t*_0_. Pore structures in soils may be represented as pore networks. For the soil formation, the network scale, *x*_0_, was proposed in [23] to be the pore separation (taken to be the median particle diameter, *d*_50_), while the rate *x*_0_/*t*_0_ was proposed [8] to be ratio of the deep infiltration rate (or subsurface run-off), ⟨*Q_sub_*〉, to the porosity. ⟨*Q_sub_*〉 turns out to be the major portion of the total run-off ⟨*Q*〉. Then, the soil production rate, *R*_s_ (represented as a function of soil depth), which is given as the derivative of the solute transport distance, is proportional to ⟨*Q_sub_*〉. In particular, $R_{s}\propto \langle Q_{sub}\rangle {\left(x/x_{0}\right)}^{-0.87}$. To define steady-state conditions, the soil production rate is then set equal to the denudation rate, *D*. The solution is,

(5) $$x=x_{0}{\left[\frac{\langle Q_{sub}\rangle }{1.87\phi D}\right]}^{1.15}$$

Here, the exponent 1.15 = 1/(1.87−1), while the numerical factor 1/1.87 arises from taking the derivative of *t* to the power 1/1.87, whereas division by *ϕ*, the porosity, converts the hydrologic flux ⟨*Q_sub_*〉 to a pore-scale velocity. ⟨*Q_sub_*〉, the subsurface run-off, is equal to ⟨*P* – *ET* – *Q_surf_*〉, where ⟨*Q_surf_*〉 is the surface run-off. Note that Equation (5), by this partitioning of run-off into surface and subsurface, is a basin-scale equation, in accordance with Budyko theory. At the local level, the infiltration rate, or vertical flow through the soil, which drives the chemical weathering and contributes to subsurface flow, would include a term with the negative of the divergence of the surface water flux. Further, *D* could be slope-dependent, although many studies assume that *D* takes on a single value throughout a given drainage basin.

For plant growth, the instantaneous transpiration rate relates a fundamental xylem diameter (similar to a pore separation) to a time scale. Both typical pore sizes and xylem diameters in porous media are on the order of 10 µm. This means that the same *x*_0_ can serve, at least approximately, as a length scale for both plant growth and soil development. Transpiration has been approximated in [22] as well as in most data collections, as *ET.* However, in [80] it was shown that the length and time scales of the mean growing season transpiration, *T*_g_, and growing season length, *t*_g_, can be used in the scaling relationship for *RRE* = *T*_g_ (*t*/*t*_g_)^1/1.21^. More importantly, since the yearly increase in *RRE* turns out to equal the transpiration [80], the root volume, which increases as the *RRE* to the mass fractal dimension, *d_f_*, of the root system, should thus be proportional to $T_{g}^{d_{f}}$. Knowing *d*_f_ is, thus, a critical input towards understanding the dependence of *NPP* on transpiration.

**Figure 2:**
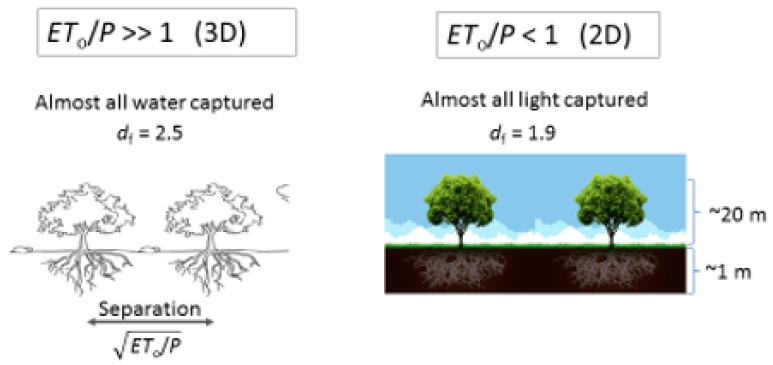
Illustration of the effects of the difference in root properties across a climate gradient in response to varying *ET*_0_/*P* (aridity index). Under more arid conditions, water is a more common factor limiting vegetation growth than are nutrients. Water is found more deeply in arid zones, while roots in forests are confined more nearly at the surface, suggesting that distinct values of the mass fractal dimensionality from percolation theory should be utilized in the two cases.

Guidance for the choice of *d_f_* was taken from percolation theory and general knowledge about the architecture of roots and the soil (see Figure 2 for details). It has often been noted that the bulk of root mass is found in the top 2 m of soil [72], or the top 1 m [73]. or even the top 0.5–0.68 m [30,74]. The top 1 m of the Earth’s surface is typically taken up by the soil [81] and the reason for the predominance of the roots in this layer is argued to trace to the concentration of nutrients in the top 1 m or so of the soil [73] Since the optimal paths exponent from percolation theory in two dimensions describes the effect of root tortuosity on the slowing in time of the increase of the root radial extent (RRE) (and thus tree height) [22,69,75,76,77], it was reasonably conjectured [22] that the mass fractal dimensionality of percolation theory in two dimensions should describe their mass as a function of a critical linear dimension (RRE). The mass fractal dimensionality of large clusters near the percolation threshold in two dimensions is 1.9; thus, *d_f_* = 1.9. It is noted that, although measured plant root fractal dimensionalities [56] tend to converge to this number (as shown in [22]), the spread in their values is quite wide. As has also been shown [26], while the percolation prediction generates nicely the global average for *ET*/*P*, measured values of *d_f_* generate the observed variability of *ET*/*P* for climatic conditions equivalent to those under which the plants were grown.

The assumption that the root mass should be proportional to the root radial extent to the 2D mass fractal dimensionality of large clusters near the percolation threshold implies that the third dimension, i.e., the root depth, is neglected. Since the thickness, *x*, of the soil layer defines rather accurately the vertical extent of the root system, the calculated 2D mass of the roots should be multiplied by the soil depth in order to generate the full productivity. Such a process will also better capture the contributions to productivity of any root symbionts, such as fungi and bacteria. Thus,

(6) $$\langle NPP\rangle =x\frac{{\langle ET\rangle }^{d_{f}}}{A_{0}}=x_{0}{\left[\frac{\langle Q_{sub}\rangle }{1.87\phi D}\right]}^{1.15}\frac{{\langle ET\rangle }^{d_{f}}}{A_{0}}$$

with *x* from Equation (5) and approximating transpiration as ⟨*ET*〉. Here, *A*_0_ is a reference area that does not depend on the fluxes and can thus be ignored in the subsequent optimization procedures.

Optimization of Equation (6) with respect to ⟨*ET*〉, with Equation (5) substituted for *x*, and ⟨*Q_sub_*〉 approximated as ⟨*Q*〉, gives 〈*ET*〉 = 0.623 ⟨*P*〉 [26]. If 1.15 is approximated as 1 and 1.9 is approximated as 2, the result is ⟨*ET*〉 = (2/3) ⟨*P*〉. The optimization is accomplished by setting *d*⟨*NPP*〉/*d*⟨*ET*〉 = 0 and leaving the denudation rate *D* in the denominator as an unknown input, independent of *ET*. The extension of theory presented here addresses the fact that the denudation rate is also a function of the hydrologic fluxes. It is important that putting together two already verified predictions (in different contexts) in terms of universal parameters yields, without adjustable parameters, the global average ⟨*ET*〉. One of these parameters, *D_b_* = 1.87, describes the time-length scaling of solute transported by advection through porous media with fundamental pore-scale connectivity in 3D.

**Figure 3:**
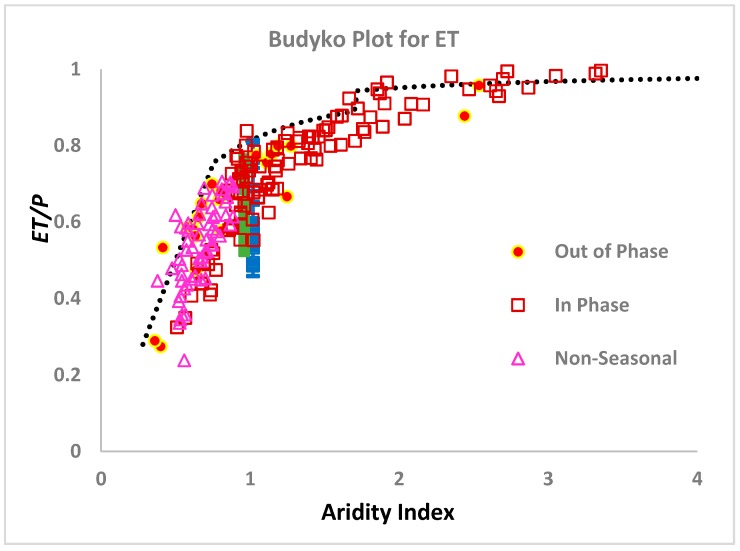
Results of *ET*/*P* plotted against aridity index. “Out of phase” and “In phase” refer to the synchronicity between maximum irradiance and precipitation. All data for *ET* were digitized from [30]. The theoretical bound was determined by the methods here. Predictions for forbs and grasses (the dominant vegetation of the northern Great Plains, [82]) were made using experimental data for root fractal dimensionality [56], rather than the percolation prediction. At large values of the aridity index, plant separation may be quite large [74], requiring an areal estimate for plant coverage.

Before moving on to the extension of the theory resulting from removing the approximations that ⟨*Q_sub_*〉 = ⟨*Q*〉 and equating transpiration with ⟨*ET*〉, two additional results already obtained should be addressed. One relates to the variability of ⟨*ET*〉 at a particular value of the aridity index, while the other focuses on the variation of ⟨*ET*〉 with aridity index. Both issues are illustrated in Figure 3.

In [26] it was pointed out that the actual root fractal dimensionality of plants was likely in many cases to deviate from the theoretical value of 1.9 obtained by [71]. One should therefore address the possibility that the range of measured ⟨*ET*〉 values might correlate with the range of predicted ⟨*ET*〉 values, if actual root fractal dimensionality values were used. For the comparison in Figure 3, data from [56] for root fractal dimensionalities of 55 forb and grass species common the Great Plains were substituted for the power 1.9 in Equation (6). Since these plants were grown under conditions of neither energy nor water limitations, the comparison is made with data for the variability of *ET* at aridity index 1, i.e., ideal conditions, although, for visibility, the representation in the figure separates the forb and grass data slightly.

At high values of the aridity index, the issue of direct evaporation cannot be neglected. Furthermore, root masses of plants, where growth is more likely to be water-limited than nutrient-limited, are more nearly isotropic, seeking water in the deeper subsurface [72,74]. Finally, it has been also shown that, in arid environments, soil depths are often not in steady-state, but still deepening in accordance with the scaling relationship for solute transport [8]. As shown in [22], all three of these inputs increase the magnitude of ⟨*ET*〉.

In desert environments, a significant fraction of the land surface can be bare [74], while plant roots are much less likely [72] to be restricted to the narrow depth range characteristic of tropical and temperate rainforests [73]. Instead of the two-dimensional percolation theoretical value of *d*_f_ for the predicted fractal dimensionality of the root system, the three-dimensional value, i.e., 2.5, should be used (Figure 2). However, if ⟨*ET*〉^2.5^ is applied, then dimensional analysis requires replacement of the factor *x* by $x^{0.5}=x^{3-d_{f}}$. In the work by Hunt [26], these changes were made in Equation (6) to address ecosystems in regions of arid climate. Further, the area covered by plants, which is known to be a decreasing function of aridity index, was assumed to be proportional to ⟨*P*〉/*ET*_0_, while ⟨*ET*〉, attributed to pure evaporation, was assumed equal to ⟨*P*〉 between plants. Substitution of *d_f_* = 2.5 into Equation (6), but using the factor *x^0.5^* yields ⟨*Q*〉 = 0.187 ⟨*P*〉, implying ⟨*ET*〉 = 0.813 ⟨*P*〉 in areas covered by plants. Then, the total ⟨*ET*〉 was written as (0.813) ⟨*P*〉 (*P*/*ET*_0_) + ⟨*P*〉 (1 − ⟨*P*〉/*ET*_0_) = ⟨*P*〉 − (0.183) ⟨*P*〉 (⟨*P*〉/*ET*_0_). Division by ⟨*P*〉 yields ⟨*ET*〉/⟨*P*〉 = 1 − 0.183 (⟨*P*〉/*ET*_0_). Consistent with the structure of this calculation, the fraction of ⟨*ET*〉 represented by evaporation from the ground surface would be (1 − ⟨*P*〉/*ET*_0_)/[1 − ⟨*P*〉/*ET*_0_ + 0.817(⟨*P*〉/*ET*_0_)] = (1 − ⟨*P*〉/*ET*_0_)/(1 − 0.183⟨*P*〉/*ET*_0_). Clearly, such a result cannot be useful when ⟨*P*〉/*ET*_0_ = 1, for which it delivers a fraction of ⟨*ET*〉 due to pure evaporation identically 0. In fact, its usefulness will be restricted more severely. Given that the global average of ⟨*E*〉/⟨*T*〉 has been suggested to be 0.36–0.39 [34,83] and that a quick estimate from the interception bounds in [61] alone generate a range of ⟨*E*〉/⟨*T*〉 values of between 18% and 73%, we infer that the use of this expression is probably limited to values of *ET*_0_/⟨*P*〉 for which it yields fractions greater than ca. 0.4. In other words, we consider evaporation of bare ground to be the primary contribution to *E* under conditions complementary to those for which evaporation off the foliage is key. For ⟨*E*〉/⟨*T*〉 = 0.4, *ET*_0_/⟨*P*〉 from the above expression is 1.575.

## 6. Extending Budyko Theory

Considering the two pathways of water, surface and subsurface, it becomes clear that the denudation rate, *D*, actually has two contributions: one from physical (subaerial) processes and one from chemical (subsurface) processes. Since, in most rivers, dissolved loads from chemical weathering are much smaller than suspended loads from physical erosion, the physical sources of erosion will dominate. The chemical erosion rate is proportional to ⟨*P*〉 − ⟨*ET*〉 − ⟨*Q_surf_*〉, whereas the physical erosion has been asserted to be proportional to ⟨*Q_surf_*〉 or, equivalently [84], to ⟨*P*〉. The first proportionality is necessary from any result that the chemical weathering rate is proportional to the flow through the soil into the subsurface. A consequence is that the chemical erosion rate is also proportional to the solute velocity, itself proportional to the flow. While it is possible to define these proportionalities, the term representing physical erosion is much less certain, making it currently inefficient to address this subject more deeply. In any case, if one makes this assumption, *D* = *a* (⟨*P* – *EP* − *Q_surf_*〉) + *b* ⟨*Q_surf_*〉, where *a* and *b* are unknown constants of proportionality. The subsurface component of run-off is typically larger than the surface component [63]. However, the fluxes in rivers from chemical weathering are typically smaller than those from physical erosion processes. Thus, *b* must normally be considerably larger than *a* (*b* ≫ *a*). Exceptions are low relief carbonate substrates, such as the Canadian shield and the St. Lawrence River that drains it.

Using the expression for *D* in Equation (5) and substituting the entire result into Equation (6) leads to

(7) $$\langle NPP\rangle =x_{0}\left(\frac{x}{x_{0}}\right)\frac{{\langle ET\rangle }^{d_{f}}}{A_{0}}=x\frac{{ET}^{d_{f}}}{A_{0}}=\frac{{\langle ET\rangle }^{d_{f}}d_{50}{\left[\frac{\langle P-ET-Q_{surf}\rangle }{1.87\emptyset \left(a\left[\langle P-ET-Q_{surf}\rangle \right]+b\langle Q_{surf}\rangle \right)}\right]}^{1.15}}{A_{0}}$$

Differentiation of ⟨*NPP*〉 with respect to ⟨*ET*〉 in Equation (7) is rather complex and was carried out in Mathematica. Nevertheless, with some approximations, it is possible to generate relatively simple expressions. For example, if one replaces 1.15 with 1 and 1.9 with 2, a quadratic equation for ⟨*ET*〉 is generated. Assuming that *b* ≫ *a*, optimization of Equation (7) yields to second order,

(8) $$\langle ET\rangle =\frac{2}{3}\left[\langle P-Q_{surf}\rangle \right]+\frac{2}{27}\frac{{\left[\langle P-Q_{surf}\rangle \right]}^{2}}{\langle Q_{surf}\rangle }\left(\frac{a}{b}\right)$$

If only the first term is retained (also consistent with *b* ≫ *a*) but the exact powers (1.9 and 1.15) are employed, the result obtained is

(9) $$\langle ET\rangle =0.623\langle P-Q_{surf}\rangle$$

Replacing the previously reported result, ⟨*ET*〉 = 0.623 ⟨*P*〉, in which the total erosion rate in Equation (5) was treated as an arbitrary input independent of the fluxes. This is the lowest order approximation that we consider.

Equation (9) predicts a smaller fraction of ⟨*P*〉 represented by ⟨*ET*〉 than our original estimate (i.e., ⟨*ET*〉 = 0.623⟨*P*〉), since the same numerical factor multiplies ⟨*P* − *Q_surf_*〉, rather than just ⟨*P*〉. However, Equation (8) makes it clear that retention of the first term by itself will lead to an underestimation of ⟨*ET*〉. Moreover, if surface run-off is included in the analysis, then so should the process of interception, < *I_t_* >, be included, since this water never makes it to the soil to be partitioned into transpiration and run-off.

Interception is viewed similarly to virga, rainfall that never makes it to the ground. Thus, interception is treated by adding an additional layer of the atmosphere within the canopy, such that only the water that penetrates the canopy is treated directly within the framework of the optimization of Equation (7). Since the partitioning at and below the ground surface is where the optimization above is performed, ⟨*l_t_*〉 is then a separate input. Define ⟨*P*〉 as the precipitation reaching the treetops. Then, it is possible to use the derivations above (Equations (7)–(9)) by substituting for ⟨*P*〉, ⟨*P*′〉 ≡ ⟨*P*〉 − ⟨*l_t_*〉, while also adding ⟨*l_t_*〉 as a separate term to evapotranspiration. Thus, to lowest order,

(10) $$\langle ET\rangle =0.623\left(\langle P-l_{t}-Q_{s}\rangle \right)+\langle l_{t}\rangle =0.623\left(\langle P-Q_{s}\rangle \right)+0.377\langle l_{t}\rangle$$

Note that the percentage of precipitation that evaporates directly from the vegetation surface is expected to be greater in areas where vegetation covers the entire surface, whereas evaporation directly from the ground surface should be reduced in that case. Therefore, the approximation in Equation (10) is likely to be most appropriate in systems where the precipitation exceeds the potential evapotranspiration, i.e., in energy-limited ecosystems. The opposite extreme of water-limited systems has already been dealt with separately.

To assess how well predictions of Equation (10) match observation, it is necessary to generate estimates of ⟨*Q_surf_*〉 and ⟨*l_t_*〉. In [45] it was estimated that 65% of ⟨*P*〉 = 834 mm is lost to ⟨*ET*〉 with 35% left for run-off. There [45] it was also indicated that 11% of the total precipitation is lost to deep infiltration. Accordingly, ⟨*Q_surf_*〉 = 0.24 *P*. In [61], [59] was cited as reporting a range of interception from 12% to 48% of precipitation. To predict to lowest order the effect of interception on total ⟨*ET*〉, one could use the midpoint (30%) of the range of ⟨*l_t_*〉 values cited above. However, [62] tabulated interception ratios from 17 studies, and determined that the mean and standard deviation of ⟨*l_t_*〉 were 18% and 10%, respectively. Using a midpoint of the range of ⟨*l_t_*〉 values cited by [61], and using Lvovitch’s value for surface run-off, Equation (10) yields 59–60% for ⟨*ET*〉. However, [63] reported a considerably higher fraction of streamflow traceable to groundwater (“*most streamflow derives from groundwater discharges, for most rivers, most of the time*”), with mean values as high as 80%. Thus, we use a range of subsurface flows that constitute between 40% and 80% of the total run-off. Fractions of ⟨*P*〉 lost to interception range [61] from 12% to 48% (a simple mean of 30%). These numbers are generally in accordance with the estimate from [34] that the worldwide average of evaporation is 39% of ⟨*ET*〉 or about 25% of ⟨*P*〉. Using 40%, 60%, and 80% for the fraction of run-off traveling through the subsurface, we report the corresponding variability in ⟨*ET*〉 due to the variability in interception in Table 1. As can be seen, the predicted ⟨*ET*〉/⟨*P*〉 values have an average of 0.645. Alternatively, we can average all 41 values for the interception component taken from [57,58,60,62]. This method gives ⟨*l_t_*〉 = 0.214⟨*P*〉 and standard deviation 0.115⟨*P*〉. Combined with the 60% run-off midpoint estimate from [63], use of a 0.214 ⟨*P*〉 value for the interception generates a mean *ET*/*P* = 0.610 ± 0.045. In this case, the variability from uncertainty in interception is not addressed, since it would merely duplicate the values already tabulated. Our best estimates for evapotranspiration thus range from 0.61 to 0.645, depending on which assessment of interception values we base the calculation on, provided we choose slightly over half (60%) of the run-off as subsurface, compatible with [63]. These values compare with the average of the pre-1995 ⟨*ET*〉/⟨*P*〉 estimates of 0.645 or the average post-1995 *ET*/*P* estimates of 0.623, for example.

Table 1 gives an estimated range of predicted fractions of ⟨*ET*〉/⟨*P*〉 based on qualitative variability in *Q_surf_* as generated from the discussion in [63], as well as from cited interception uncertainty. The first three entries are generated from the general bounds on interception cited by [61]. The last entry is generated from the mean and standard deviation of the data summarized in [57,58,60,62]. Note, however, that one should probably expect a correlation between surface fluxes and interception, which means that uncorrelated Gaussian statistical analyses should not be relied on for quantitative predictions.

The first three entries use values for interception from [61] and a variability in subsurface run-off fraction compatible with [63]; the last entry uses the mean and standard deviation of the experimental values compiled from [57,58,60,62], and the middle value for subsurface run-off fraction compatible with [63]. Application of estimates for the partitioning of surface and subsurface run-off from [45] would reduce the predicted ⟨*ET*〉/⟨*P*〉 ratio further; in the first case to about 0.58, and in the second to about 0.55.

The tendency of ⟨*ET*〉/⟨*P*〉 to approach 1 in the limit of large aridity index is seen clearly in Figure 3. At a very large aridity index, our prediction of ⟨*ET*〉/⟨*P*〉 derived from the steady-state soil depth expression (Equation (6)) underestimates ⟨*ET*〉. However, it has been shown [9] that, in this limit, steady-state is seldomly achieved and soils are still deepening. Use of the time-dependent scaling function for soil depth generates a different dependence on ⟨*Q*〉 (more nearly the square root of ⟨*Q*〉), and shifts the balance even further toward ⟨*ET*〉. This predicted change to non-steady state behavior in the soil depth produces the sudden jump of predicted ⟨*ET*〉 values around the aridity index of 1.8. However, it is likely that a more gradual increase should be expected; thus, the choice of aridity index 1.8 for the cross-over amounts to use of an adjustable parameter.

## 7. Discussion: Variability and Discrepancies

Since it has been possible to generate an expected global average of ⟨*ET*〉 in accordance with observations, the question arises as to what causes the variability in ⟨*ET*〉 at a given aridity index. It was already shown in [26] that the actual fractal dimensionality of root systems of characteristic species of the Great Plains could be used to predict the variability in ⟨*ET*〉, at least at aridity index 1. Now, it appears that such factors as the partitioning of ⟨*ET*〉 into ⟨*E*〉 and ⟨*T*〉, as well as the partitioning of run-off into subsurface and surface run-off can also introduce significant variability into the predictions. These various sources of variability can be incorporated into the model, but in different ways. Nevertheless, a quantitative estimate based on the magnitudes of predicted spread in their values of ⟨*ET*〉/⟨*P*〉 (0.06, 0.07, and 0.16) for the variability due to uncertainty in run-off, interception, and root fractal dimensionality, can be made. The R^2^ values that result are: 0.06^2^/(0.06^2^ + 0.08^2^ + 0.16^2^) = 0.08, 0.08^2^/(0.06^2^ + 0.08^2^ + 0.16^2^) = 0.15, and 0.162/(0.6^2^ + 0.08^2^ + 0.16^2^) = 0.76 for their contributions to the variability in ⟨*ET*〉. Such an analytical estimation is possible only if other potential inputs are neglected and the statistics are Gaussian. The run-off characteristics will relate most strongly to variability in soil type and depth, as well as climatic variables that affect precipitation intensity and regularity, while interception characteristics appear to relate most strongly to seasonality of precipitation and tree canopy physical structure. Root architecture appears to be the most important single variable, when plant species are considered individually. Presumably, when entire ecosystem response is addressed, however, variability in root structure from assemblages of plants would be reduced relative to individual plant species, at least when those ecosystems are not disturbed.

In Figure 3, we also note that, for large aridity index, all the observed values of ⟨*ET*〉/⟨*P*〉, which are higher than our predicted curve, have maximum precipitation in-phase with maximum radiation, while the opposite is true in the energy-limited regime, where precipitation and radiation, which are in phase, are most likely to be well below the limiting value. In the energy-limited case, then, all of the values of ⟨*ET*〉/⟨*P*〉 above the predicted limit are for systems where ⟨*P*〉 and *R* maxima are out of phase, or where precipitation is non-seasonal. This result is in close correspondence to the statement of [85] “when water and energy supplies are out of phase, observed mean annual evapotranspiration is lower than the amount predicted in the absence of seasonality, while it is higher when water and energy supplies are in phase”. Although the interpretation of in-phase systems is likely quite complicated, a relatively simple explanation may be available for the out-of-phase systems. In particular, consider a system with aridity index 1, but for which energy and water supplies are out of phase. Optimization of water for soil formation and vegetation growth simultaneously is not possible, since energy is not available when the water is. Thus, some additional water runs off. Consequently, the relevant energy supply is smaller, and an effective aridity index would be smaller than the actual aridity index.

We should also discuss the unique role of ⟨*Q_surf_*〉 in the optimization of ⟨*NPP*〉. ⟨*Q_surf_*〉 is one of the smaller fluxes in magnitude, but its effect on the landscape is one of the most intense. Furthermore, in contrast to the other hydrologic fluxes, its effect is highly variable, in both space and time. Knowledge of this variability is important for a wide range of scientific and practical applications: evaluation of the surface characteristics, such as local slope, infiltration, groundwater recharge, soil hydraulic conductivity, antecedent moisture contents, and vegetation health, as well as because a very large fraction of the denudation of the landscape takes place during intense weather events. Thus, ⟨*Q_surf_*〉 has a disproportionately large role in landscape evolution, while its input is also highly variable as well as quantitatively unpredictable. This combination of factors should lead to the treatment of ⟨*Q_surf_*〉 as a stochastic input process, with potentially important non-linear contribution. In the present context, these complex attributes of surface water fluxes make it difficult to characterize their effects on the role of vegetation in the landscape, particularly as concerns an optimization of productivity.

In our discussion thus far, we tacitly assume that the variability in measured values of interception as well as in the relative fractions of surface and subsurface run-off is reliably due to distinct conditions in different experiments in distinct ecosystems with different climatic variables. It is also possible that some of the variability is measurement error or lack of closure of water budgets. While we cannot evaluate the magnitude of this uncertainty, the possibility that newer measurements may force revision of existing estimates cannot be simply put aside.

## 8. Time Scales for Ecosystem Adjustment to Changes in External Parameters, Such as in Climate or Land-Use

At the outset, it should be stated that the current theoretical framework cannot predict such important time scales as plant dispersal into newly suitable habitats. Perhaps it will be possible in the future to incorporate known plant dispersal rates into our existing model. Nevertheless, it is possible to combine use of our theory and collected data to predict time scales for the development and maturation of soil.

**Figure 4:**
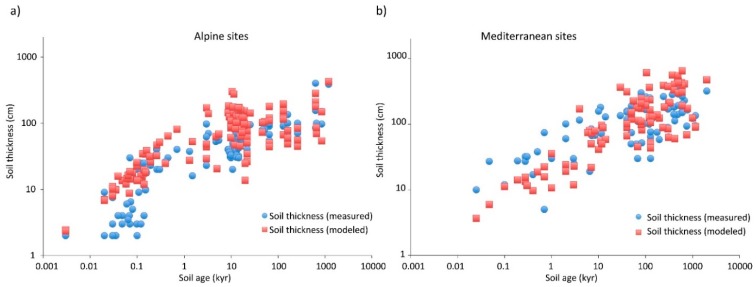
After [23]. A demonstration of the ability of percolation concepts to predict actual soil depths. Note, however, the distinction between alpine (**a**) and Mediterranean (**b**) sites. In the former case, soil depths at time scales up to about 200 years may be overpredicted. The interpretation is that in some cases reaction kinetics provided a stronger limitation on the weathering process than transport. note that the upper limit of these soil depths has slope approximately one, rather than one-half, consistent with a time-independent weathering rate. Figure 4a thus illustrates a time scale that defines equal influences on chemical weathering from reaction kinetics and solute transport (just over 100 years).

Soil development to a particular depth appears in many cases to be defined purely by the physical process of solute transport in its limitation of chemical weathering. Nevertheless, there are some cases where solute transport does not appear to be the limiting factor in soil development, as shown in Figure 4. Such cases appear to combine slow reaction kinetics with high flow rates and coarse particles. A unitless ratio of the advection time to the reaction time, the Damköhler number, *Da_I_* = *τ_A_*/*τ_R_* [10,64], is useful for assessing the relative importance of solute transport and reaction kinetics to reaction rates in porous media. As long as *Da_I_* ≫ 1, solute transport is limiting; the other limit, when *Da_I_* ≪ 1, is kinetics limited. When a reaction is limited by the kinetics, to lowest order (neglecting changes in the nature of mineral surfaces), the reaction rate is constant. When soil formation is limited by a reaction rate that is constant, the soil formation rate should be constant and the soil depth increase linearly in time. The details in the calculation of *Da_I_* make this subject beyond the present scope (for further details, see [10,23]). However, the consequences of a soil formation rate that is constant can be seen in Figure 4, where, at short time scales, some alpine soil depths increase linearly in time and more slowly than predicted purely on the basis of transport limitations. Note that estimations of *Da_I_* for these cases [23] suggested that its value increased from less than 1 to greater than 1 at length scales of about 10 cm, roughly in accordance with the data, which exhibit a cross-over to transport limitations at a compatible length scale. In the following, since transport limitations appear dominant in the formation of most soils, we discuss biological and denudation time scales in comparison with time scales derived from transport limitations.

We can define a time scale for erosion, *t_D_*, from the ratio of the soil depth to the denudation rate, *x*/*D*. The time scale for soil formation to a depth *x* is $t_{x}=t_{0}{\left(x/d_{50}\right)}^{D_{b}}$. Equating *t_x_* with *t_0_* = *d_50_ Φ*/*Q_sub_*, leads almost to Equation (5), but without the factor 1.87 in the denominator. This small discrepancy arises from the fact that Equation (5) is derived by setting the denudation and soil formation rates equal, which involves the derivative of the soil depth as a function of time. Use of such time scales as defined in this section, however, allows determination of the time required to achieve steady state depth, although not necessarily steady-state soil characteristics. Consider that the thicknesses of natural soils are virtually always within an order of magnitude of 1 m. *t_D_* ranges can be easily calculated from the ratio of 1 m to the denudation rate, *D*, a quantity which ranges from a minimum of about 1 m/Myr in arid continental interiors, such as in Australia, or deserts such as the Atacama, to about 1000 m/Myr in tectonically active regions with high precipitation, such as the New Zealand Alps or the Himalayan mountain range. Thus, *t_D_* ranges from about 1 Myr to 1 kyr. For soils of a few decimeter thickness, this time scale can be as small as about 200 years. Attainment of steady-state soil depths at compatible time scales can be seen in a range of environments from the California Central Valley to Gongga Mountain in China in the work of [8], marked by cessation of deepening of soils. In the work of [23], denudation rates between 50 m/Myr and 20 m/Myr correspond to calculated time scales for attainment of steady-state from about 20 kyr to about 50 kyr, respectively. In [23], alpine soils require about 20 kyr, but Mediterranean soils closer to 100 kyr than 50 kyr to reach steady state depth.

**Figure 5:**
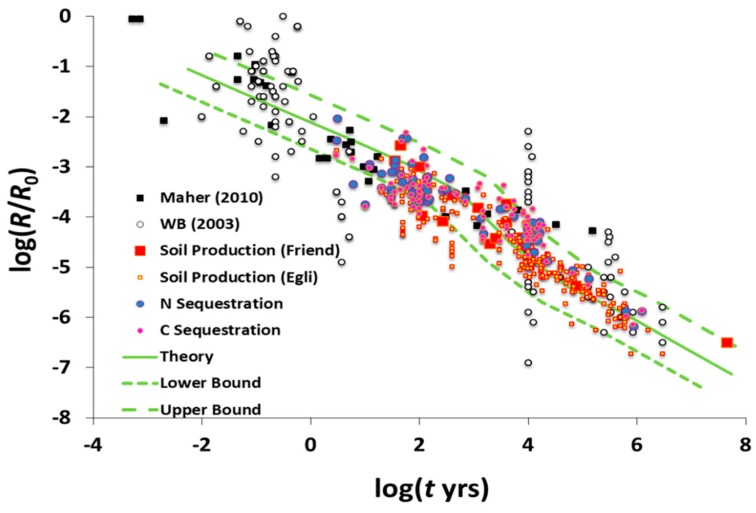
Prediction of chemical weathering rates for strongly heterogeneous media using flow rate variability ±1 order of magnitude (after [11]).

Soil health or productivity is related to its concentration of carbon and of nutrients. At very short times and small soil depths, the slowing of solute transport from particle sources is still relatively ineffective in limiting soil deepening. Thus, on time scales of up to a few decades, rather remarkably large soil depths can be attained, up to decimeters. The rate of accumulation of carbon can be roughly constant, in accordance with perennial vegetation growth rates of decimeters to about a meter per year. However, as fractional carbon content in the soil approaches a steady-state value and as the rate of soil deepening slows, the possibility that soil production could limit the rate of increase of stored carbon emerges (cf. Figure 5 (after [11])). Note that the soil formation (or production) rate follows our prediction very closely over the entire range of observed time scales. However, the soil carbon and nitrogen sequestration rates remain relatively constant for periods up to nearly a century, before they start to decay in accordance with prediction. This may be interpreted as a sign that the nutrient accumulation rate at any particular site is roughly constant until the soil reaches a kind of nutrient carrying capacity. After this point, it is possible to increase total nutrients in the soil only if the soil depth increases. This time period for adjustment appears, according to Figure 5, to be on the order of 100 years, at least for the suite of soils studied.

Because the time to reach a steady-state soil depth is, ordinarily, much longer than the time for ecosystems to reach an approximate steady-state, it is possible to evaluate the soil depth dependence in the soil nutrient content over very long-time scales. For soils with extremely long time-scales for reaching steady-state (in the case of deep tropical weathering), the limitation of nutrient access may be critical. There, weathering depths may be so great that fresh mineral nutrients are supplied at rates so low that a completely different nutrient limitation is important. When times of erosion for 1 m of soil approach 1 Myr, soil production rates slow accordingly, setting free mineral nutrients at rates that hinder biological productivity. In such cases, it does not really matter what factors reduce soil production rates correspondingly; if it is the great depth to the weathering front, nutrient limitations will hinder plant productivity, but, if it is the slow rate of water flow into the ground (in extremely arid regions), the lack of water will hinder productivity even more fundamentally. Since soil formation rates are approximately inversely proportional to soil depth but approximately proportional to subsurface run-off, a three orders of magnitude reduction in precipitation rates (e.g., from the New Zealand Alps to interior Australia) will produce a three orders of magnitude reduction in soil formation just as will a three order of magnitude increase in weathering depth (from, e.g., coastal California to the Amazon rainforest).

## 9. Conclusions and Recommendations

Within the framework of percolation theory, we investigate effects of incorporating separately surface and subsurface run-off into Budyko formalism for the water balance. Since we are unable to predict these finer distinctions, what is proposed is merely the development of an equation that can account for additional information. In the original calculation of ⟨*ET*〉/⟨*P*〉, it was found that run-off constituted a little over 1/3 of *P* and evapotranspiration a little less than 2/3 of ⟨*P*〉. Our new findings are that: (1) accounting for the distinction between transpiration and interception tends to increase the fraction of ⟨*P*〉 going to ⟨*ET*〉; (2) accounting for the distinction between surface and subsurface run-off tends to reduce the fraction of ⟨*P*〉 going to ⟨*ET*〉; and (3) the net effect of accounting for both tends to leave the fraction going to ⟨*ET*〉 nearly unchanged, although possibly slightly reduced, depending on which information regarding approximate values of infiltration and subsurface flows was accessed. This result appears to make our framework more adaptable to interfacing with numerical models and observations, which can better deliver such quantities as interception, since this value is not predictable within our theoretical framework. In principle, the separation of effects of interception and evaporation off bare ground from the transpiration component allow a prediction of the variability of CO_2_ drawdown from the atmosphere along climate gradients, which would not have been possible when these results were lumped together into evapotranspiration.

Further, we cannot show it likely that the impact of root architecture on the fundamental partitioning is a more important source of variability in the evapotranspiration data than either the variable partitioning of interception vs. transpiration or surface vs. subsurface flow. If this result can be extended across climate zones, then it should be possible to generate analytical predictions of the changes in the interactions between the carbon and water cycles with climate change using chiefly the variability in root fractal dimensionality to generate the variability in <*ET*>.

Our results are based on the relevance of chemical weathering to soil formation and the tendency for chemical weathering to be limited by advective solute transport. Thus, it is the weathering that is slowed by the transport, not the transport that is slowed by the weathering! The exponent dictating this slowing is the universal scaling of solute transport times versus system length as given in percolation theory. Past work has shown that the percolation exponent (backbone mass fractal dimensionality in 3D, under saturated conditions) generates the observed temporal dependence of weathering rates as well as the global average of the overall partitioning of precipitation into run-off and evapotranspiration. Currently, we also show that these fractions are mostly maintained when distinctions between surface and subsurface run-off as well as between interception and transpiration are accounted for.

Our results imply the desirability of addressing several factors in greater detail: (1) determine the values of the constants *a* and *b* relating denudation rates to the subsurface and surface run-off fluxes, respectively; (2) consider the implications of phase differences in the seasonality of precipitation and energy fluxes; (3) incorporate, as a function of aridity index, the role of the variability of actual root fractal dimensionality on the water balance through its effects on productivity; and (4) apply the framework to individual basins, for which all the input parameters are known, aridity index, interception, and the constants that describe the relationships of chemical and physical erosion to the fluxes.

## Figures and Tables

**Table 1:** Predicted ⟨***ET***〉/⟨***P***〉 values and their variabilities due to uncertainty in interception.

Subsurface Fraction of Run-Off	Predicted ⟨*ET*〉/⟨*P*〉	Variability from Interception
0.4	0.59	0.068
0.6	0.642	0.068
0.8	0.691	0.068
0.6	0.610	0.045
